# Systemic mastocytosis with *KIT* V560G mutation presenting as recurrent episodes of vascular collapse: response to disodium cromoglycate and disease outcome

**DOI:** 10.1186/s13223-017-0193-x

**Published:** 2017-04-24

**Authors:** Iolanda Conde-Fernandes, Rita Sampaio, Filipa Moreno, José Palla-Garcia, Maria dos Anjos Teixeira, Inês Freitas, Esmeralda Neves, Maria Jara-Acevedo, Luis Escribano, Margarida Lima

**Affiliations:** 1 Consulta Multidisciplinar de Linfomas Cutâneos e Mastocitoses (CMLC), Hospital de Santo António (HSA), Centro Hospitalar do Porto (CHP), Porto, Portugal; 2 Serviço de Dermatologia, Hospital de Santo António (HSA), Centro Hospitalar do Porto (CHP), Porto, Portugal; 3 Serviço de Anatomia Patológica, Hospital de Santo António (HSA), Centro Hospitalar do Porto (CHP), Porto, Portugal; 4 Laboratório de Citometria, Serviço de Hematologia Clínica, Hospital de Santo António (HSA), Centro Hospitalar do Porto (CHP), Ex-CICAP, Rua D. Manuel II, s/n, 4099-001 Porto, Portugal; 5 Serviço de Hematologia Laboratorial, Hospital de Santo António (HSA), Centro Hospitalar do Porto (CHP), Porto, Portugal; 6 Serviço de Imunologia, Hospital de Santo António (HSA), Centro Hospitalar do Porto (CHP), Porto, Portugal; 7 Unidade Multidisciplinar de Investigação Biomédica, Instituto de Ciências Biomédicas da Universidade do Porto (UMIB/ICBAS/UP), Porto, Portugal; 8 Servicio General de Citometría, Instituto de Biología Molecular y Celular del Cáncer, Centro de Investigación del Cáncer, Salamanca, Spain; 9 Departamento de Medicina, Universidad de Salamanca (IBMCC-CSIC/USAL), Salamanca, Spain; 10 Spanish Network on Mastocytosis (REMA), Toledo, Spain

**Keywords:** Mast cells, Systemic mastocytosis, Mast cell activation disorders, Recurrent anaphylaxis, *KIT* V560G mutation, Disodium cromoglycate

## Abstract

**Background:**

Mastocytosis are rare diseases characterized by an accumulation of clonal mast cells (MCs) in one or multiple organs or tissues. Patients with systemic mastocytosis (SM), whose MCs frequently arbor the activating D816V *KIT* mutation, may have indolent to aggressive diseases, and they may experience MC mediator related symptoms. Indolent SM with recurrent anaphylaxis or vascular collapse in the absence of skin lesions, ISMs(−), is a specific subtype indolent SM (ISM), and this clonal MC activation disorder represents a significant fraction of all MC activation syndromes. The V560G *KIT* mutation is extremely rare in patients with SM and its biological and prognostic impact remains unknown.

**Case presentation:**

A 15-year old boy was referred to our hospital because of repeated episodes of flushing, hypotension and syncope since the age of 3-years, preceded by skin lesions compatible with mastocytosis on histopathology that had disappeared in the late-early childhood. Diagnosis of ISM, more precisely the ISMs(−) variant, was confirmed based on the clinical manifestations together with increased baseline serum tryptase levels and the presence of morphologically atypical, mature appearing (CD117+high, FcεRI+) phenotypically aberrant (CD2+, CD25+) MCs, expressing activation-associated markers (CD63, CD69), in the bone marrow. Molecular genetic studies revealed the presence of the *KIT* V560G mutation in bone marrow MCs, but not in other bone marrow cells, whereas the screening for mutations in codon 816 of *KIT* was negative. The patient was treated with oral disodium cromoglycate and the disease had a favorable outcome after an eleven-year follow-up period, during which progressively lower serum tryptase levels together with the fully disappearance of all clinical manifestations was observed.

**Conclusions:**

To the best of our knowledge this first report of a patient with ISM, whose bone marrow MCs carry the *KIT* V560G activating mutation, manifesting as recurrent spontaneous episodes of flushing and vascular collapse in the absence of skin lesions at the time of diagnosis, in whom disodium cromoglycate had led to long term clinical remission.

## Background

Mastocytosis are rare diseases characterized by an accumulation of clonal mast cells (MCs) in one or multiple tissues, whose clinical manifestations mostly depend on the overall MC burden, the organs and/tissues involved, and the effects of the MC mediators such as histamine, leukotrienes, tryptase and heparin [1].

Criteria for the diagnosis and classification of mastocytosis were discussed extensively by the experts on the subject and consensus proposals were made in the last years [2, 3]. These proposals were adopted by the World Health Organization (WHO) in 2001 [4] and 2008 [5], and lastly updated in 2016 [6]. The 2016 classification of the WHO divides the disease into cutaneous mastocytosis (CM), systemic mastocytosis (SM), and localized mast cell tumors [6, 7]. Cutaneous mastocytosis includes maculopapular CM, also known as urticaria pigmentosa, diffuse CM, and localized mastocytoma of skin. Systemic mastocytosis is further divided into indolent SM, smoldering SM, and advanced SM variants, including aggressive SM, mast cell leukemia (MCL), and SM with associated hematologic neoplasm (SM-AHN), previously known as SM with associated clonal hematologic non-MC lineage disease (SM-AHNMD) [6, 7]. Other rare SM variants have been described so far, such as well-differentiated SM (WDSM) [8, 9] and ISM with recurrent anaphylaxis or vascular collapse in the absence of skin lesions, ISMs(−) [10].

The diagnosis of SM is based on the identification of neoplastic MCs by morphological, immunophenotypic, and genetic studies in organs and/or tissues outside the skin, including mostly the bone marrow (BM), and requires at least one major and one minor criteria, or three minor criteria [4–7]. The only major criterion of SM is the multifocal accumulation and clustering of MC (at least 15 MC/cluster) in the BM or another extra-cutaneous organ. Minor criteria include an abnormal MC morphology, expression of CD2 and/or CD25 in MC in extracutaneous organs, an activating mutation in codon 816 of *KIT* (usually *KIT* D816V) in extra-cutaneous cells, and a basal serum tryptase level exceeding 20 ng/ml. Monitoring of serum tryptase is useful both for the diagnosis and follow-up of the disease [11].

Indolent SM with recurrent anaphylaxis or vascular collapse in the absence of skin lesions, ISMs(−), is a specific subtype of clonal MC activation disorder (cMCAD) [12, 13], and represents a significant fraction of all MC activation syndromes (MCAS), a heterogeneous group of conditions in which the most prominent clinical manifestations result from the release of MC mediators [14, 15]. The European Competence Network on Mastocytosis (ECNM) classified MCAS into three major groups: primary (corresponding to cMCAD), secondary (allergy related), and idiopathic (no clonal MCs and MCA triggers identified) [14]. Patients with cMCAD can be further subdivided into those fulfilling the WHO criteria for the diagnosis of SM and those satisfying only one or two minor criteria [14].

Recently, we reviewed the clinical and laboratory features of a series of adult patients with mastocytosis referred to and followed in a dedicated multidisciplinary consultation for mastocytosis in our center [16]. Here we describe in detail one of these patients with recurrent episodes of vascular collapse, who presented a rare *KIT* V560G activating mutation in bone marrow MCs, and had a very favorable outcome after a follow-up of 11-year, with a remarkable clinical and laboratory response to oral disodium cromoglycate (DSCG).

## Case report

**Fig. 1:**
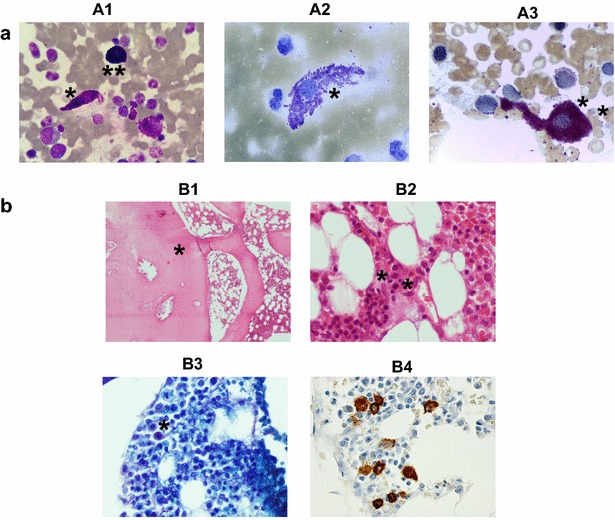
Bone marrow cytological and histopathological features at diagnosis. **a** Leishman’s (*A1*), toluidine blue (*A2*) and chloroacetate esterase (*A3*) staining of bone marrow (BM) smears from the patient at diagnosis, revealing atypical mast cells (MCs) with elongated cytoplasmic extensions and abnormal granulation (*). A normal MC is shown for comparison (*A1*, **). **b** Hematoxilin-eosin (*B1*, 40×; *B2*, 400×), Giemsa staining (*B3*, 400×) and CD117 immunostaining (*B4*, 400×) of a BM trephine biopsy from the patient at diagnosis, showing normocellular marrow with osteosclerosis (*B1*, *) and scattered elongated and degranulated abnormal (*B2* and *B3*, *), CD117 positive (B4) MCs

**Fig. 2:**
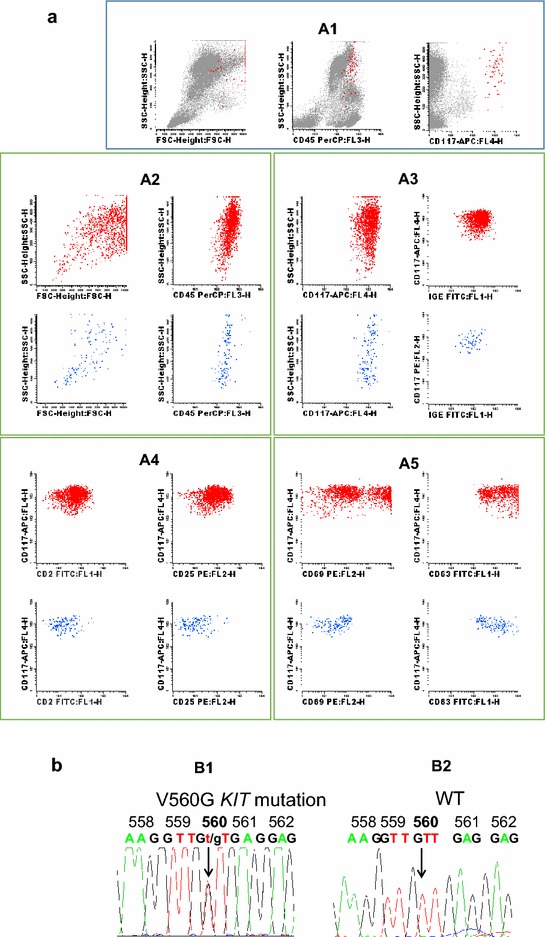
Immunophenotypic and genetic features of bone marrow (BM) mast cells (MCs). **a** Illustrating bivariate dot-plots of the BM cells from the patient (*panel A1*), and after selecting specifically for BM mast cells (*panels A2*–*A4*); as illustrated, bone marrow MCs showed a mature (CD117+high, FcεRI+high) (*panel A2*), aberrant (CD2+, CD25+) (*panel A3*) immunophenotype, with expression of activation-associated markers (CD63, CD69) in a large fraction of the cells (*panel A4*). Corresponding *dot plots* from normal bone marrow MCs (*blue dots*) are show for comparison. **b** Sequences of polymerase chain reaction (PCR) products illustrating the presence of the *KIT* V560G mutation in bone marrow MCs from the patient, at diagnosis (*panel B2*); the wild-type (WT) sequence is shown for comparison

A 15 year-old Caucasian male was referenced to our institution in November 2005 for investigation of severe episodes of flushing, hypotension and vascular collapse since his late-early childhood; at that time, he referred a past history of several admissions at the emergency room for similar episodes. He had no history of urticaria, angioedema, and allergy or identified MCA triggers. In particular, the life-threatening episodes were not caused by food, medications, insect bites, temperature changes, exercise, or other recognized factors. Of note, the patient had been diagnosed with cutaneous mastocytosis at the age of 3-year, based on histopathological analysis of maculopapular skin lesions which disappeared soon thereafter. At the time of referral, he didn’t have skin lesions, hepatomegaly, splenomegaly, or lymphadenopathy, and he was under H1 anti-histamines therapy, without the relief of the crises. Laboratory investigation showed markedly increased baseline serum tryptase levels (175 μg/l; normal range: 2–13 µg/l). Transient increase in serum tryptase above the baseline values during the MC-mediators related events were not documented, because blood samples for determination of serum tryptase levels were not taken at the episodes flushing, hypotension and vascular collapse. The BM aspirate was normocellular with increased numbers of MC (1.7%), most of which had an atypical (type I) morphology (spindle-shaped MC with irregular distribution and focal accumulation of the cytoplasmic granules, and elongated cytoplasmic extensions); the myeloid/erythroid ratio was normal and there was no myelodysplasia (Fig. 1, panel A). The BM trephine (stained with hematoxylin-eosin and Giemsa), showed a normocellular BM without dysplastic features or increased blast cells, but a few scattered morphologically abnormal MCs, difficult to recognize (Fig. 1, panel B). Dense BM mast cell aggregates (>15 cells/aggregate) were not observed. Flow cytometry confirmed the presence of BM mast cells with abnormally high light scatter values (SSC and FSC), and a mature (CD117+high, FcεRI+high), but aberrant (CD2+, CD25+) and activated (CD63+high, CD69+high) phenotype (Fig. 2, panel A). Molecular analysis showed the absence of the *KIT* Asp-816(r)Val mutation; in contrast, the *KIT* Val-560(r)Gly activating mutation was detected in FACS-purified BM mast cells (Fig. 2, panel B), but not in other BM myeloid and lymphoid cells, including BM precursors. Serum IgE levels were within normal values (7 KU/l; normal levels <100 KU/l), and the screening for IgE antibodies specific for common food and inhalant allergens and Hymenoptera venom was negative. The remaining work-up, which included a full blood cell count, biochemical tests, bone densitometry and abdominal ultrasound, was unremarkable. Thus, although the patient had no skin lesions since his late childhood, the case fulfilled the WHO criteria for the diagnosis of ISM. Based on these findings and on the clinical manifestations, the patient was diagnosed with the ISMs(−) variant [12, 13]. H1 anti-histamines were maintained and oral DSCG (200 mg capsules, administered 5-times/day) was added. Since the start of DSCG therapy, systemic MC-associated symptoms have remained under control and serum tryptase levels progressively decreased over time, from 175 µg/l in November 2005 (first observation) to 61 µg/l in 2008, 49 µg/l in 2011, 45 µg/l in 2013, 31 µg/l in August 2015 and 35 µg/l in September 2016 (last observation). The patient is currently 26 years-old and he remains completely asymptomatic under DSCG therapy.

## Methods

Serum tryptase levels were measured by using the UniCAP tryptase fluoroenzyme immunoassay (Pharmacia Diagnostics AB, Uppsala, Sweden). The presence of IgE antibodies to the mixtures of common inhalant and food allergens was screened with the ImmunoCAPT™ Phadiatop Infant^®^ (Pharmacia Diagnostics AB, Uppsala, Sweden).

Flow cytometry immunophenotyping of BM mast cells was performed using a stain-lyse-and-then-wash direct immunofluorescence technique (BD FACS Lysing Solution™, Becton Dickinson Biosciences, BD, CA, USA), accordingly to the recommendations of the REMA [17]. Briefly, BM cells were stained with 4-color combinations of monoclonal antibodies directed against the CD45, CD117, CD2, CD25, CD63, CD69 and FcεRI, conjugated with fluorescein isothiocyanate (FITC), phycoerythrin (PE), peridinin chlorophyll protein (PerCP) or allophycocyanin (APC). Anti-CD45 (PerCP) and anti-CD117 (APC) were combined in different tubes with anti-IgE (FITC)+anti-CD33 (PE), anti-CD2 (FITC)+anti-CD25 (PE), or anti-CD63 (FITC)+anti-CD69 (PE). Data acquisition was performed in a FACSCalibur flow cytometer (BD), using the BD CellQuest™ software (BD). In the first step, 50,000 events from the whole BM cells were acquired per tube, and CD117+ high BM mast cells, were quantified. In the second step, acquisition though a live gate specifically drawn to include only BM mast cells was performed and only the events included in this region were stored. For data analysis, the Infinicyt™ software (version 17.0, Cytognos, Salamanca, Spain) was used. Mast cells were first identified based on their typical CD45+CD117+high phenotype and then characterized for the expression of other cell surface markers.

*KIT* mutations were detected in highly purified (>97% purity) BM cell populations as previously described in detail [18]. Positivity for the *KIT* V560G mutation was confirmed by sequencing the PCR products in both directions in an ABI Prism 3130 Genetic Analyzer (Applied Biosystems, Foster City, CA), using the 5′-CCA GAG TGC TCT AAT GAC TG-3 and 5-AGC CCC TGT TTC ATA CTG AC-3 primers (Isogen Life Sciences) and the dye-deoxy terminator method.

## Discussion

The case here reported fulfills the WHO criteria for the diagnosis of SM, with three minor diagnostic criteria being present: increased baseline serum tryptase levels, morphologically atypical and phenotypically aberrant (CD2+, CD25+) bone marrow MCs [6, 7]. However, in contrast to what is observed in most of the patients with ISM, the *KIT* D816V mutation was absent [19, 20]. Instead, the BM mast cells, but not other BM cells, were found to have the *KIT* V560G activating mutation. Interestingly, while skin lesions compatible with mastocytosis had occurred at the age of 3-year, they had regressed in the late-early childhood, the only disease-associated symptoms that persisted over time being recurrent episodes of flushing, hypotension and vascular collapse.

Clonal MC activation disorders comprise a heterogeneous group of conditions that do not always fulfil the WHO criteria for SM; such conditions typically shave gain of function *KIT* mutations associated to constitutively activated MCs [12–15]. The clinical manifestations may vary in type and severity, and they usually include abdominal cramping, diarrhea, tachycardia, arrhythmias, irritability, and/or many other signals and symptoms related to the release of MC mediators. As it occurred in our patient, life-threatening episodes of anaphylaxis, including flushing, hypotension and vascular collapse, taking place spontaneously or after a Hymenoptera sting, may occur, particularly in patients who show no skin lesions typical of mastocytosis [12–15].

Systemic mastocytosis has been associated with the presence of somatic mutations in the v-kit Hardy-Zuckerman four feline sarcoma viral oncogene homolog (*KIT*), which encodes for a transmembrane protein stem cell receptor with kinase activity (KIT receptor, CD117) that binds to the stem cell factor (SCF) [21, 22]. Typically, in the majority (>90%) of the ISM patients the A71763T substitution is detected at codon 816 in exon 17 of the *KIT* gene, whereby an aspartate is changed for a valine of the KIT protein sequence [21, 22]. However, other uncommon somatic (e.g., V560G, D815K, D816Y, D816F, D816H, and D820G) and germline (e.g., F522C, A533D, K509I and del419) mutations have been reported in a small number of cases [21, 22]. Of note, this is the first case described so far in the literature who carried the *KIT* V560G activating mutation.

Two activating point mutations of *KIT* leading to two distinct amino acid substitutions in the KIT receptor, i.e. Asp-816(r)Val and Val-560(r)Gly, have been described in the MC leukemia cell line (HMC-1), derived from a patient with SM [21–23]. The D816V mutation affects the activating loop of the KIT receptor and occurs in >90% of adult SM patients [18–21, 24], not only in the BM, but also in the peripheral blood [18, 25]. In contrast, the V560G mutation involves the juxta membrane domain of the KIT receptor and is preferentially found in gastrointestinal stromal tumors, been reported in only a few SM cases [19, 20, 26–28] (Table 1). In this regard, it should be noted that despite the first description of the V560G *KIT*-mutation has been made decades ago, this mutation was not found in purified BM mast cells from a series of 113 adult patients diagnosed with SM at the reference centers of the Spanish Network on Mastocytosis (REMA) [19], published in 2006. Interestingly, however, it was subsequently described to be present in BM mast cells from 1 of 123 patients (0.8%) in a subsequent update of the REMA 2006 series, published in 2010 [20]. In parallel, the *KIT* V560G mutation was found in the skin biopsy of only 1 CM patient out of 142 adult patients diagnosed with mastocytosis at the reference centers of the French Mastocytosis Network (AFIRMM) [28], which confirms that this is a rare but recurrent *KIT* mutation in mastocytosis.

**Table 1:** Frequency of *KIT* codon 816 and 560 mutations in patients with mastocytosis

Reference	Büttner et al. [26]	Yanagihori et al. [27]	Garcia-Montero et al. [19]	Teodosio et al. [20]	Lanternier et al. [28]
Country	Germany	Japan	Spain	Spain	France
Number of patients studied	17	16	113	123	142
Adults/children	6/11	12/4	113/0	123/0	142/0
Males/females	NA	12/4	58/55	66/57	43/93
CM/SM	17 (100%)/0 (0%)	16 (100%)/0 (0%)	0 (0%)/113 (100%)	0 (0%)/123 (100%)	38 (27%)/104 (73%)
Adult/childhood onset	6 (35%)/11 (65%)	4 (25%)/12 (75%)	NA/NA	NA/NA	114 (80%)/28 (20%)
Sample types tested	Skin	Skin	Bone marrow	Bone marrow	Skin
Cell types tested	All cells	All cells	Mast cells	Mast cells	All cells
*KIT* codon 816 mutations	6/17 (35%)	14/16 (88%)	102/113 (90%)	93/123 (6%)	97/138 (70%)
Adult onset	6/6 (100%)	4/4 (100%)	NA	NA	86/112 (77%)
Children onset	0/11 (0%)	10/12 (83%)	NA	NA	11/26 (42%)
*KIT* codon 560 mutations	2/6 (33%)	0/16 (0%)	0/113 (0%)	1/123 (0.8%)	1/138 (0.7%)
Adult onset	2/4 (50%)	0/4 (0%)	NA	NA	1/112 (0.9%)
Children onset	0/2 (0%)	0/12 (0%)	NA	NA	0/26 (0%)

*CM* cutaneous mastocytosis, *SM* systemic mastocytosis, *NA* not available

Previous studies have emphasized the relevance of performing molecular studies in purified BM mast cells, as well as in other BM cells, such as CD34+ precursors and mature myeloid and lymphoid BM cells to search not only for the *KIT* D816V mutation, but also for other *KIT* mutations, particularly if the BM mast cells have an aberrant immunophenotype and/or abnormal morphological features [19, 20]. In accordance, Garcia-Montero et al. [19] have showed the presence of *KIT* D816V mutation in virtually all adults (93%) with SM, while other *KIT* mutations were rarely (<3%) detected. In addition, they demonstrated that in around one-third of patients with SM, the *KIT* mutation was detected not only in MC, but also in CD34+ hematopoietic cells and eosinophils, and, to a lesser extent, in monocytic and neutrophil-lineage BM precursor cells and lymphocytes [19]. Furthermore, they found that most patient with poor-prognosis SM (81%) carried the *KIT* mutation in two or more BM myeloid cell populations, while this occurred in a smaller proportion (27%) of indolent SM cases [19]. Moreover, a number of studies have suggested that “atypical” (non D816V) *KIT* mutations may facilitate transformation to more advanced MC disease [7]; however, the prognostic value of the *KIT* V560G activating mutation is not established, due to its rarity. Additional mutations in other genes (e.g., TET2, SRSF2, ASXL1, CBL, RUNX1, and RAS, and less commonly, JAK2 V617F and RUNX1-RUNX1T1) may occur in patients suffering from aggressive SM, MCL or SM-AHN, ASM, or MCL [7].

The activating *KIT* D816V and V560G mutations, despite targeting the same receptor, have a distinct impact on downstream signaling pathways, at the same time they might potentially be targeted by different tyrosine kinase inhibitors (TKI) [23]. Thus, the Janus kinase 3/signal transducer and activator of transcription (JAK3/STAT) pathway is the preferred signaling pathway involved in the *KIT* V560G mutation, whereas the mammalian target of rapamycin complex 1/4E-binding protein 1 (mTORC1/4E-BP1) pathway is preferentially linked to the *KIT* D816V mutation [23].

The *KIT* D816V mutation, which is found in the majority of patients with SM and initiates a number of signal transduction events, is thought to trigger abnormal MC activation. However, not all patients with *KIT* D816V + SM suffer from MC activation symptoms, suggesting that other mechanism may be implicated. Unfortunately, till recently the only in vitro model available for studying the impact of *KIT* mutations on human MC biology was the HMC-1 cell line, which arbor the *KIT* V560G mutation. Two sublines of HMC-1 were subsequently described, named HMC-1.1 (*KIT* V560G+) and HMC-1.2 (*KIT* V560G+, *KIT* D816V+) [29]. However, the fact that both HMC-1 sublines contain the *KIT* V560G mutation, whereas only the HMC-1.2 subline exhibits the *KIT* D816V mutation, makes it difficult to compare the signaling pathways activated by each of the mutant forms of KIT. To overcome these limitations, Saleh et al. [30] have recently established a *KIT* D816V+ SCF-independent MC line (ROSAKIT D816V), for which a KIT SCF-dependent wild type (ROSAKIT WT) exists. They found that ROSAKIT D816V cells produce a MC tumors in mice models, but, unexpectedly, ROSAKIT D816V cells exhibited a decreased responsiveness to IgE-dependent stimuli as compared to ROSAKIT WT cells, and mice with ROSAKIT D816V-derived MC tumors did not show MC mediator-related symptoms. In addition, they realized that *KIT* D816V+ MC obtained from patients with SM did not show increased IgE-dependent histamine release. They concluded that *KIT* D816V mutation does not activate MC to release proinflammatory mediators, and they proposed that MC mediator-related symptoms in patients with SM occur preferentially in the context of a coexisting allergy [30]. Our patient, whose BM mast cells harbor the *KIT* V560G mutation, had MC-mediator related events without clinical or laboratory evidence of allergy, as documented by normal serum IgE levels and negative screening for IgE antibodies specific for food and inhalant allergens. Thus, it would be of interest to explore whether *KIT* V560G promotes, instead of suppressing, IgE-mediated histamine release.

Overall, three distinct MC immunophenotypic profiles (mature, activated and immature) were described so for patients with SM, which correspond to different disease subtypes, namely WDSM, ISM/cMCAD and ASM/MCL, respectively [20]. So, BM mast cells from patients with ISM and cMCAD have a high light scatter, a mature (CD117+high, FcεRI+high), activated (overexpression of CD63, CD69, CD203c, and other activation related markers) and aberrant (CD25+, CD2+) phenotype, as found in this case.

Symptomatic treatment of SM is mainly based on avoidance of factors triggering the release of MC mediators and may also include H1 and H2 anti-histamines, proton pump inhibitors, anti-leukotrienes, anti-cholinergic, corticosteroids, DSCG, and/or epinephrine in case of anaphylactic episodes. In aggressive SM, treatment with interferon alpha, cladribine (2-chlorodeoxyadenosine) or TKIs should to be considered. Concerning TKIs, imatinib mesylate is effective only in patients who do not carry the *KIT* D816V mutation or other exon 17 mutations [31]. The patient here reported, showing limited tumor burden and life-threatening episodes of vascular collapse, required only symptomatic therapy.

Cromoglicic acid (also known as cromoglycate or cromolyn), which is marketed as a sodium salt (disodium cromoglycate, DSCG, or cromolyn sodium), belongs to the group of chromones and is a mast cell stabilizer that prevents the release of MC mediators [32, 33]. Its mechanism of action remains poorly understood [32, 33]. DSCG has minimal systemic absorption following ingestion and negligible fat solubility, suggesting an inability to enter the cells and an interaction with a cell surface receptor [32, 33]. In permeabilized mast cells, DSCG inhibits GTP-gamma-S-induced secretion by a mechanism not involving the nucleoside diphosphate kinase [34]. In activated neutrophils DSCG selectively inhibits O^2^—generation but not degranulation and such effect may be related with inhibition of assembly of an active NADPH oxidase and prevention of oxygen radical-induced tissue damage [35]. Inhibition of MC-mediated immediate-type hypersensitivity by DSCG has also been reported [36]. In addition, DSCG was found to be a G-protein-coupled receptor 35 agonist [37]. However, the exact biochemical basis of the response of MC carrying activating *KIT* mutations to DSCG are not known and it would be interesting to investigate if the KIT receptor itself or downstream signaling pathway components may serve as targets for this MC stabilizer.

Disodium cromoglycate is available as nasal spray (for allergic rhinitis), eye drops (for allergic conjunctivitis), and oral forms (capsules and aqueous solution, used to treat urticaria, ulcerative colitis, and mastocytosis), although availability varies from one country to another. Cromolyn sodium, capsules (100 mg) and oral solution concentrate (100 mg/5 ml), was approved by the Food and Drug Administration for the treatment of mastocytosis in adults and children aged more than 2 years-old, being licensed for that in the United States (capsules and ampules) and in Canada (capsules). In that concerning the effectiveness of DSCG in treatment of mastocytosis, the randomized controlled trials performed are scarce (we have found only four studies published in journals indexed in MedLine), historic (reported from 1979 to 1990), and small (enrolling 5–13 patients/trial); they have used variable doses of DSCG (200 mg taken orally four times daily in most cases) in different formulations (capsules, tablets or oral aqueous solution) and the results obtained were not totally concordant [38–41]. In the late 70s, a double-blind crossover study of the efficacy of oral DSCG was carried out in 5 patients with SM for periods of 8–32 months; the authors observed that DSCG (but not placebo) produced marked amelioration of the pruritus, flushing, diarrhea, abdominal pain and disorders of cognitive function [38]. In the 90s, a double-blind, placebo-controlled trial of the efficacy of oral DSCG was conducted in 11 patients with SM; this study showed that DSCG was significantly helpful relative to placebo in alleviating the gastrointestinal manifestations, whereas the benefit for non-gastrointestinal symptoms did not reach statistical significance [39]. In between these studies, two other trials were published. In one of them, 13 patients (3 children and 10 adults) with mastocytosis having cutaneous and systemic symptoms participated in a blind trial and were treated for 1 month with placebo and for another month with oral DSCG; it was found that most patients experienced amelioration of the pruritus, improvement in urtication and relief of gastrointestinal manifestations when treated with DSCG [40]. The other study compared the efficacy of oral DSCG and combined H1 and H2 antihistamines [41]. In this study, 8 patients with SM were randomly assigned to receive either DSCG + placebo or antihistamines + placebo, with crossover at the end of week 10, and only 6 patients completed the study; although the differences between the two treatments were not statistically significant, the authors noted that most patients had less pruritus and less urticaria while receiving antihistamines, while most patients had improvement of the gastrointestinal symptoms and one patient experienced dramatic relief from bone pain while receiving DSCG [41]. In addition, case reports and small series of patients have stated benefits in using oral and topical DSCG for the treatment of diffuse cutaneous/bullous mastocytosis [42–44]. Moreover, oral and inhaled DSCG have been used in the management of other symptoms associated with SM, such as of bone pain, headache and fatigue, and hypotensive crisis [45, 46]. Despite the low level of evidence found in the literature, DSCG is one of the mainstays in the symptomatic treatment of SM, being recommended by the experts from centers where a large number of patients with mastocytosis are followed, to alleviate gastrointestinal, cutaneous, neuropsychiatric, skeletal and other systemic disease related symptoms [47–49].

In Portugal, DSCG is not approved for the treatment of mastocytosis and the drug is not available in oral formulations. Our patient was treated off-label with manipulated gelatinous capsules containing 200 mg DSCG; as described, this therapeutic approach successfully abrogated the clinical manifestations related to the release of MC mediators, and might therefore be a non-toxic treatment option for this very small and unique subset of SM patients. Because childhood onset mastocytosis often regresses spontaneously by puberty, the possibility of the disappearance of the clinical manifestations in our patient being due to spontaneous regression of the disease with the advancing age cannot be completely excluded. However, this hypothesis seems improbable: first, when the patient was first observed in our consultation he had already 15 years old and he still had very high basal serum tryptase levels (13 times the upper limit of normal) and frequent episodes of flushing, hypotension and vascular collapse; second, there was a clear relationship between the beginning of DSCG treatment and the control of the clinical manifestations, with no such episodes being observed thereafter. In addition, although basal serum tryptase levels have declined steadily over the years after starting DSCG therapy, they were still increased at the last observation (2.6 times the upper limit of normal), indicating that the disease remains active, although clinically controlled.

In summary, here we described a young patient with SM whose the BM mast cells were found to harbor the activating *KIT* V560G mutation, in association with a mature, activated, and aberrant phenotype, who showed a sustained complete response of MC activation-associated life-threatening symptoms with long term therapy with DSCG.

## Conclusions

Patients with SM, whose MCs frequently arbor the activating D816V *KIT* mutation, may have indolent to aggressive diseases, and they may experience diverse types of MC mediator symptoms. In contrast, the V560G mutation, which uses different signaling pathways to promote MC growth, is extremely rare and its biological and prognostic impact remains unknown. Here we reported a patient with recurrent spontaneous episodes of severe flushing and vascular collapse, who harbor the V560G *KIT* mutation in mature activated aberrant BM mast cells, in whom DSCG treatment had led to long term clinical remission.

## Abbreviations

AFIRMM: French Mastocytosis Network
APC: allophycocyanin
ASM: aggressive systemic mastocytosis
BD: Becton–Dickinson
BM: bone marrow
CM: cutaneous mastocytosis
DSCG: disodium cromoglycate
cMCAD: clonal mast cell activation disorder
ECNM: European Competence Network on Mastocytosis
FITC: fluorescein isothiocyanate
ISM: indolent systemic mastocytosis
ISMs(−): indolent systemic mastocytosis without skin involvement (associated with recurrent anaphylaxis or vascular collapse)
JAK3/STAT: Janus kinase 3/signal transducer and activator of transcription
KIT: v-kit Hardy-Zuckerman 4 feline sarcoma viral oncogene homolog
MC: mast cell
MCAS: mast cell activation syndrome
MCL: mast cell leukemia
mTORC1/4E-BP: mammalian target of rapamycin complex 1/4e-binding protein 1
PE: phycoerythrin
PerCP: peridinin chlorophyll protein
REMA: Spanish Network on Mastocytosis
SCF: stem cell factor
SM: systemic mastocytosis
SM-AHNMD: systemic mastocytosis with an associated clonal hematological non–mast cell disorder
TKI: tyrosine kinase inhibitor
WDSM: well-differentiated systemic mastocytosis
WHO: World Health Organization
